# iTRAQ-Based Comparative Proteomic Analysis of the Roots of TWO Winter Turnip Rapes (*Brassica rapa* L.) with Different Freezing-Tolerance

**DOI:** 10.3390/ijms19124077

**Published:** 2018-12-17

**Authors:** Xiucun Zeng, Yaozhao Xu, Jinjin Jiang, Fenqin Zhang, Li Ma, Dewei Wu, Youping Wang, Wancang Sun

**Affiliations:** 1 College of Agronomy and Biotechnology/Key Laboratory of Hexi Corridor Resources Utilization of Gansu, Hexi University, Zhangye 734000, China; xiucunzeng@126.com (X.Z.); xuyaozhao@126.com (Y.X.); fenqinzh@hxu.edu.cn (F.Z.); 2 College of Agronomy, Gansu Agricultural University, Lanzhou 730070, China; 18189560623@163.com; 3 Jiangsu Provincial Key Laboratory of Crop Genetics and Physiology, Yangzhou University, Yangzhou 225009, China; jjjiang@yzu.edu.cn (J.J.); dewei@yzu.edu.cn (D.W.)

**Keywords:** *Brassica rapa*, turnip, differentially abundant proteins, freezing stress

## Abstract

The freezing tolerance of roots is crucial for winter turnip rape (*Brassica rapa* L.) survival in the winter in Northwest China. Cold acclimation (CA) can alleviate the root damage caused by freezing stress. To acknowledge the molecular mechanisms of freezing tolerance in winter turnip rape, two *Brassica rapa* genotypes, freezing stressed after the induction of cold acclimation, were used to compare the proteomic profiles of roots by isobaric tags for relative and absolute quantification (iTRAQ). Under freezing stress (−4 °C) for 8 h, 139 and 96 differentially abundant proteins (DAPs) were identified in the roots of “Longyou7” (freezing-tolerant) and “Tianyou4” (freezing-sensitive), respectively. Among these DAPs, 91 and 48 proteins were up- and down-accumulated in “Longyou7”, respectively, and 46 and 50 proteins were up- and down-accumulated in “Tianyou4”, respectively. Under freezing stress, 174 DAPs of two varieties were identified, including 9 proteins related to ribosome, 19 DAPs related to the biosynthesis of secondary metabolites (e.g., phenylpropanoid and the lignin pathway), and 22 down-accumulated DAPs enriched in oxidative phosphorylation, the pentose phosphate pathway, fructose and mannose metabolism, alpha-linolenic acid metabolism, carbon fixation in photosynthetic organisms, ascorbate and aldarate metabolism. The expressional pattern of the genes encoding the 15 significant DAPs were consistent with the iTRAQ data. This work indicates that protein biosynthesis, lignin synthesis, the reduction of energy consumption and a higher linolenic acid content contribute to the freezing tolerance of winter turnip rape. Functional analyses of these DAPs would be helpful in dissecting the molecular mechanisms of the stress responses in *B. rapa*.

## 1. Introduction

Freezing stress can lead to metabolic blocks, the destruction of cell membrane integrity, and even death in plants due to the intracellular ice formation in plant tissues. However, plant response to stress is always active. To survive, plants have formed various protective mechanisms to improve their freezing tolerance [1]. Of them, the freezing tolerance of plants can be enhanced in periods of low nonfreezing temperatures. This enhancement is called cold acclimation (CA). CA can induce the production of antifreeze proteins and freezing-related gene expressions [1,2]. Many freezing tolerance genes, such as the early response to dehydration 7 (*ERD7*), heat shock cognate protein gene (e.g., *HSC70-1*), have been identified in plants by transcriptome analysis [3], which greatly contribute to the understanding of the freezing tolerance of plants. Although these data are very useful, mRNA expression levels do not directly correspond to the protein abundance [4,5]. Protein expression relies on many factors, such as the transcription rate of genes, transcript stability, translational regulation and protein degradation [5]. In addition, proteins are the basic molecular practitioners of life activities and control the ultimate biological processes. Thus, proteomics can provide more direct information on cell metabolisms’ response to abiotic stresses [6]. The isobaric tags for relative and absolute quantification (iTRAQ) is a high-throughput proteomic technique that allows for the simultaneous identification and quantification of numerous proteins in different samples [7,8]. Recently, iTRAQ has been applied to study the proteomic profile and cold-stress-responsive proteins in plants [9,10,11].

Winter turnip rape (*Brassica rapa* L.) is a valuable oil crop, broadly planted in Northwest China for its high-quality edible oil and capability of conserving soil and water [12]. However, long and extreme cold weather in Northwest China negatively influences the growth and development, overwintering, and propagation of the winter turnip rape. Before the overwintering period, the aboveground part of the winter turnip rape shrivels, and the root is its only organ for overwintering. Therefore, the freezing tolerance of roots decides whether the plant will survive the winter. As is well known, CA plays a very important role in alleviating the damage caused by adverse environmental factors, and the CA of winter turnip rape roots is crucial for increasing root freezing tolerance. Recently, we identified the cold-stress-responsive microRNAs (miRNAs) in *Brassica rapa* by high-throughput sequencing and enriched the knowledge of microRNA-mediated cold responses at the posttranscriptional level [13]. However, direct protein information about the root freezing tolerance of *B. rapa* is still lacking. In this study, two winter turnip rape varieties, ‘Longyou7’ (freezing-tolerant) and ‘Tianyou4’ (freezing-sensitive), were used to analyze the root proteomic changes in response to freezing stress after the induction of CA. We found that the freezing tolerance of winter turnip rape was related to the protein biosynthesis, improvement of the cell wall thickness, linolenic acid content, and the alleviation of sugar consumption caused by the suppression of energy and carbohydrate metabolism, which will provide a basis for the molecular mechanism study of the freezing tolerance of plants with different genotypes. 

## 2. Results

### 2.1. Analysis of Physiological Parameters under Freezing Stress

**Figure 1:**
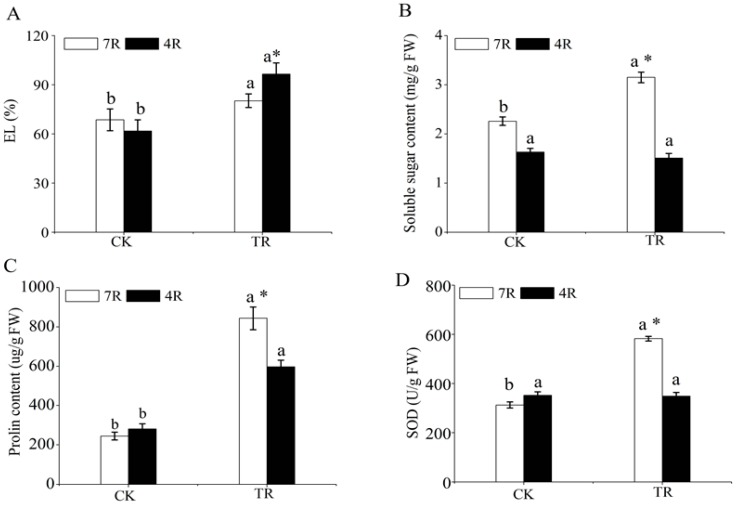
Responses of physiological parameters under freezing stress. (**A**) Variations in electrolyte leakage. (**B**) Soluble sugar content. (**C**) Proline content. (**D**) Superoxide dismutase (SOD) activity. Control (CK) refers to the control treatment at 20 °C; TR represents the freezing treatment at −4 °C; 7R and 4R represent the winter turnip rape varieties, Longyou 7 and Tianyou 4, respectively; the mean and SD were calculated from three repeats of each treatment; the bars indicate the standard deviation; the columns marked with different letters (a and b) indicate significant statistical differences among the CK and TR of two varieties based on Ducan’s multiple range tests (*p < 0.05). The column marked with an asterisk indicates the significant difference in treatment (TR) of two varieties based on an independent-samples *t*-test (*p < 0.05).

The electrolyte leakage (EL), soluble sugar content, proline content and superoxide dismutase (SOD) activity in the roots of “Longyou7” (7R, freezing-tolerant) and “Tianyou4” (4R, freezing-sensitive) were analyzed (Figure 1). Compared with the control (CK), the level of EL and proline content were significantly increased in the freezing-stressed 7R (7RTR) and 4R (4RTR) (Figure 1A,B), and the soluble sugar content and SOD activity were significantly increased in 7R, but no significant difference was observed in 4R (Figure 1C,D). Under freezing treatment (TR), the EL level of 7R was significantly lower than that of 4R, but other physiological indexes of 7R were higher than those of 4R. These results indicated the roots of 7R with higher tolerance to freezing stress than those of 4R.

### 2.2. Primary Data Analysis and Protein Identification by iTRAQ

**Figure 2:**
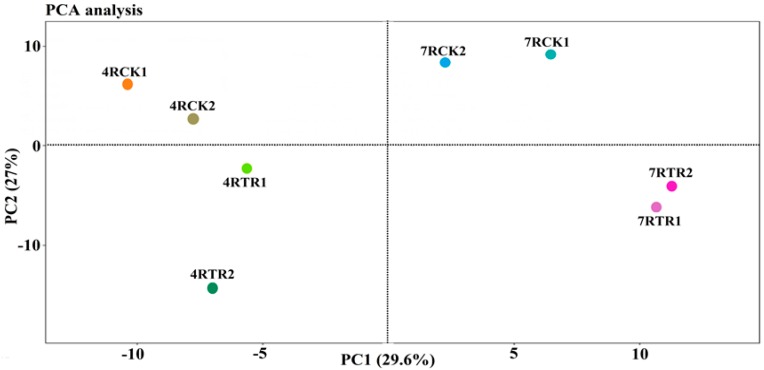
Principle component analysis (PCA) of the proteome from the freezing-stressed and non-stressed winter turnip rape roots. 7RCK and 7RTR denote CK and TR treatments of the R7 variety, respectively. 4RCK and 4RTR denote CK and TR treatments of the R4 variety, respectively.

In this study, the root samples for iTRAQ analysis were named, 4RCK (root of “Tianyou4” at 20 °C), 4RTR (root of “Tianyou4” treated at −4 °C), 7RCK (root of “Longyou7” at 20 °C), and 7RTR (root of “Longyou7” treated at −4 °C). Based on the iTRAQ analysis, 268,959 spectra were generated from the roots of two winter turnip rape varieties using iTRAQ. A total of 38,122 spectra were matched to the known spectra, 15,611 spectra were matched to the unique spectra, 11,666 mapped peptides, 9706 mapped unique peptides, and 2724 mapped proteins, with a 0.6% false discovery rate (FDR) (Figure S1 and Table S1, Supplementary Materials). The length and number distribution of the peptides as well as the mass and sequence coverage of the proteins are provided in Figure S2 (Supplementary Materials). The principal component analysis (PCA) showed that two biological replicates of each sample had good repeatability (Figure 2). The repeatability analysis of two biological replicates also showed a protein coverage of more than 90%, based on a 50% variation in each sample (Figure S3, Supplementary Materials) which demonstrated the reliability of the proteomics analyses, according to a previous study [7]. Furthermore, PCA found that there was a great difference between 7RTR and 7RCK as well as that between 4RTR and 4RCK (Figure 2), which showed that freezing stress greatly changed the abundance and quantity of proteins in winter turnip rape. 

### 2.3. Identification and Gene Ontology (GO) Annotation of Differentially Abundant Proteins (DAPs) under Freezing Stress

**Figure 3:**
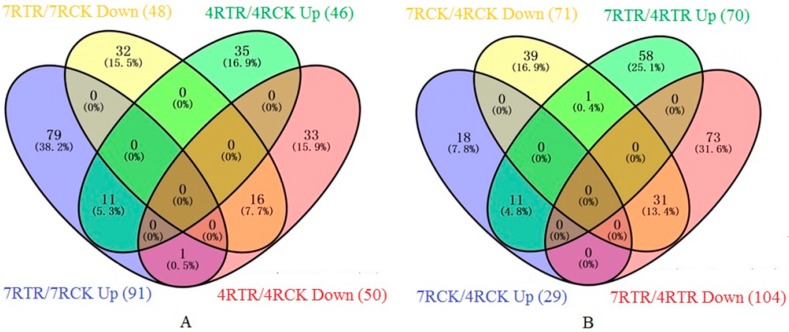
Venn diagrams of the differentially abundant proteins (DAPs) of different comparison groups. (**A**) Venn diagrams of the DAPs between 7RTR and 7RCK as well as those in 4RTR and 4RCK. (**B**) Venn diagrams of the DAPs between 7RTR and 4RTR as well as those in 7RCK and 4RCK. 7RTR/7RCK is the protein abundance ratio of 7RTR to 7RCK, 4RTR/4RCK is the protein abundance ratio of 4RTR to 4RCK, 7RCK/4RCK is the protein abundance ratio of 7RCK to 4RCK, and 7RTR/4RTR is the protein abundance ratio of 7RTR to 4RTR.

The DAPs were determined according to the ratio with more than a 1.5-fold or less than a 0.67-fold change, and the *p*-value is less than 0.05. In this study, 139 DAPs between 7RTR and 7RCK were identified (Table S2, Supplementary Materials), including 91 up- and 48 down-accumulated proteins. Ninety-six DAPs were identified between 4RTR and 4RCK (Table S3, Supplementary Materials), including 46 up- and 50 down-accumulated proteins. Among these DAPs, we identified 11 common proteins that were up-accumulated in both 7RTR/7RCK and 4RTR/4RCK, and 16 common proteins that were down-accumulated both in 7RTR/7RCK and 4RTR/4RCK (Figure 3A), implying that these common DAPs are stable and responsive to freezing stress. One hundred and seventy-four DAPs were identified between 7RTR and 4RTR, including 70 up- and 104 down-accumulated proteins (Table S4, Supplementary Materials). A comparison of the DAPs between two varieties indicated that 11 proteins were common under stressed and non-stressed conditions, which were up-accumulated in both 7RCK/4RCK and 7RTR/4RTR. Moreover, 31 common proteins were down-accumulated, in both 7RCK/4RCK and 7RTR/4RTR (Figure 3B). Some specific DAPs between 7RTR and 7RCK, 4RTR and 4RCK, and some DAPs with a high fold change between 7RTR and 4RTR are summarized in Table 1, and the detailed lists are provided in Table S1 (Supplementary Materials). 

**Figure 4:**
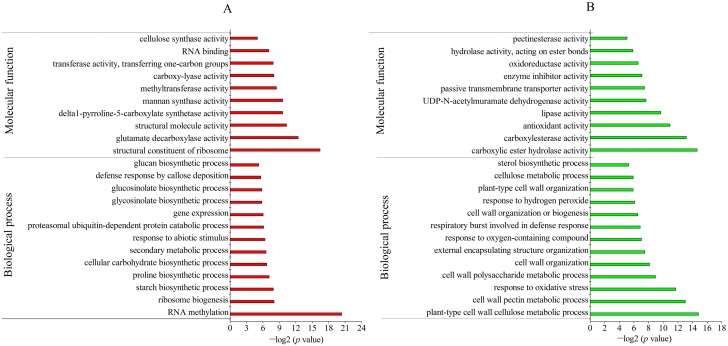
Gene ontology (GO) enrichment analysis of up- and down-accumulated DAPs between 7RTR and 7RCK. (**A**) Up-accumulated proteins. (**B**) Down-accumulated proteins.

Gene ontology (GO) analysis revealed the biological process and molecular function of DAPs between TR and CK. For the biological process, most of the up-accumulated DAPs in the freezing-stressed 7R were involved in RNA methylation, ribosome biogenesis, and the starch and proline biosynthetic process. Besides, some stress-related GO terms were also enriched, including the response to abiotic stimulus, gene expression and defense response by callose deposition. Under the category of molecular functions, these up-accumulated DAPs were mostly enriched in the structural constituent of ribosome, glutamate decarboxylase activity, and structural molecule activity (Figure 4A). Interestingly, delta1-pyrroline-5-carboxylate synthetase was involved in proline biosynthesis, which was also enriched with DAPs. The down-accumulated DAPs were mainly involved in the cell wall metabolic process, including the plant-type cell wall cellulose metabolic process, cell wall pectin metabolic process and cell wall polysaccharide metabolic process. In the category of molecular functions, these down-accumulated DAPs were mostly involved in carboxylic ester hydrolase activity, carboxylesterase activity, antioxidant activity and lipase activity (Figure 4B).

**Figure 5:**
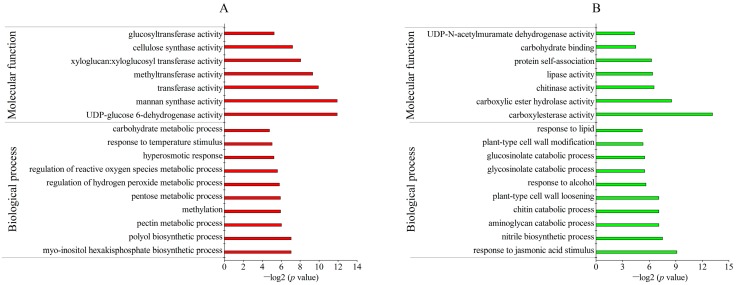
GO enrichment analysis of up- and down-accumulated DAPs between 4RTR and 4RCK. (**A**) Up-accumulated proteins; (**B**) down-accumulated proteins.

Under freezing stress, most of the up-accumulated DAPs of 4R participated in the response to the myo-inositol hexakisphosphate biosynthetic process, polyol biosynthetic process and pectin metabolic process. Under the category of molecular functions, these up-accumulated DAPs were mainly related to uridine diphosphate (UDP)-glucose 6-dehydrogenase activity, mannan synthase activity and transferase activity (Figure 5A). In the biological process, the down-accumulated DAPs of 4R mainly participated in the response to jasmonic acid stimulus, nitrile biosynthesis and the aminoglycan catabolic process. As for the molecular function, most of the down-accumulated DAPs were related to carboxylesterase activity, carboxylic ester hydrolase activity, chitinase activity and lipase activity, which was similar to the functions of down-accumulated DAPs in freezing-stressed 7R (Figure 5B).

### 2.4. Kyoto Encyclopedia of Genes and Genomes (KEGG) Pathway Enrichment Analysis of DAPs between Two Freezing-Stressed Varieties 

**Figure 6:**
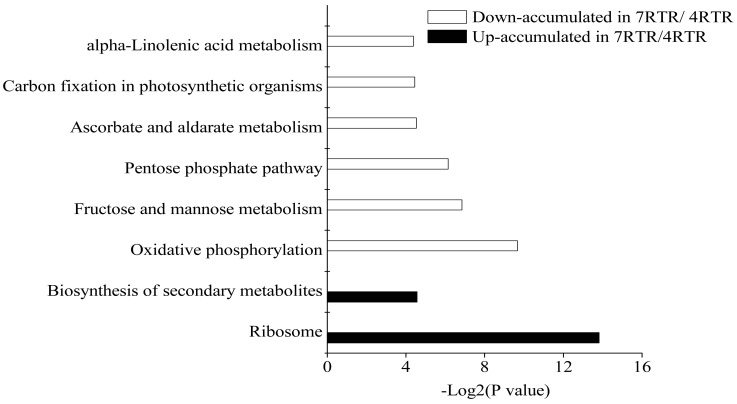
Kyoto Encyclopedia of Genes and Genomes (KEGG) pathway enrichment analysis of DAPs between 7RTR and 4RTR.

**Figure 7:**
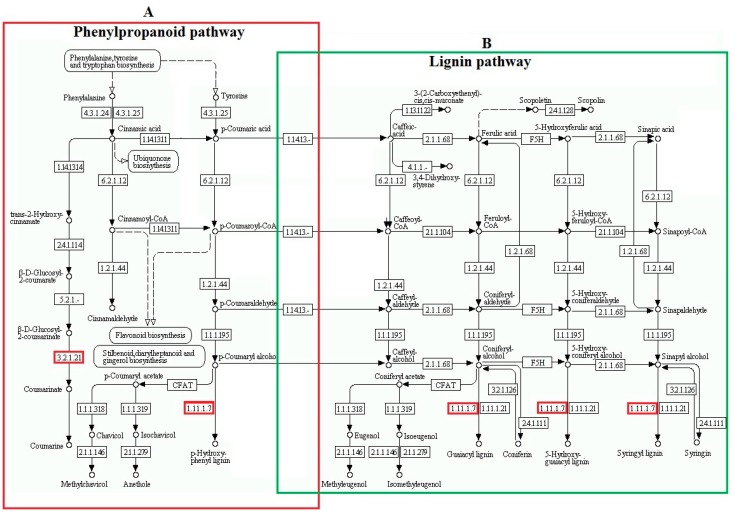
DAPs involved in phenylpropanoid and the lignin pathway of winter turnip rape. (**A**) Phenylpropanoid pathway. (**B**) Lignin pathway. The colored enzyme codes are noted as follows: Gly-Asp-Ser-Leu (GDSL) esterase/lipase (EC: 3.2.1.21), Peroxidase C3 and Peroxidase A2 (EC: 1.11.1.7).

The Kyoto Encyclopedia of Genes and Genomes (KEGG) pathway enrichment analysis was conducted to determine the biological pathways involved in the DAPs between 7RTR and 4RTR (Figure 6; Table S5, Supplementary Materials). We found 22 down-accumulated DAPs in 7RTR/4RTR, which were significantly enriched in oxidative phosphorylation (path: ko00190), the pentose phosphate pathway (path: ko00030), fructose and mannose metabolism (ko00051), ascorbate and aldarate metabolism (path: ko00053), carbon fixation in photosynthetic organisms (path: ko00710) and alpha-linolenic acid metabolism (path: ko00592). Nine and 19 up-accumulated DAPs in 7RTR/4RTR were significantly enriched in ribosome (path: ko03010) and the biosynthesis of secondary metabolites, respectively. To acknowledge the biosynthesis of secondary metabolites responsive to freezing stress, we analyzed the DAPs involved in related pathways based on GO analysis and the protein expression level. Three up-accumulated DAPs in 7RTR/4RTR were related to phenylpropanoid and the lignin pathway (path: ko00940) (Figure 7). 

### 2.5. Validation of Genes-Encoding DAPs by qRT-PCR

**Figure 8:**
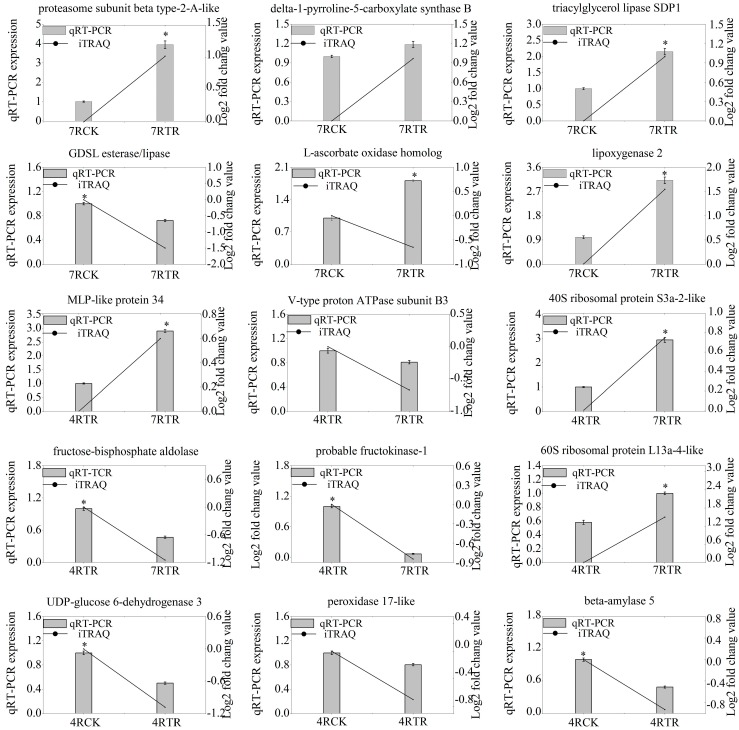
Analysis of the transcript levels of DAPs by a quantitative real-time polymerase chain reaction (qRT-PCR). The candidate genes were from differential protein genes between 7RTR and 7RCK, between 7RTR and 4RTR, and between 4RTR and 4RCK. Statistically significant differences (Duncan’s multiple range tests, * *p* < 0.05) of transcription analysis by qRT-PCR are indicated by asterisks.

To elucidate the correspondence between mRNA and protein expression patterns, we performed a transcription analysis of 15 representative DAPs by qRT-PCR (Figure 8). The result showed that the qRT-PCR data of 14 genes agreed with the iTRAQ results, such as the Gly-Asp-Ser-Leu (GDSL) esterase/lipase-like protein (GELP), major latex like protein 34 (MLP 34), and beta-amylase 5. On the other hand, a gene-encoding L-ascorbate oxidase homolog showed the opposite expressional pattern to the protein levels, which might be due to the post-transcriptional regulation, such as mRNA turnover, translation rate, and/or post-translational protein stability.

## 3. Discussion

The selection and breeding of freezing tolerance cultivars is of great importance to production of winter turnip rape in northwestern regions. The overwinter survival rate is a direct index that evaluated freezing tolerance of winter turnip rape, and more than 70% overwinter survival rate is an indicator of its safety overwintering [14]. A previous study found that at an extreme natural low temperature (−32 °C), the overwinter survival rate of “Longyou7” was more than 90%, but “Tianyou4” could not survive at the extreme low temperature and its survival rate was 62.4% overwinter at −9 °C [14]. Therefore, according to the overwinter survival rate of winter rate, “Longyou7” and “Tianyou4” were evaluated as freezing-tolerant and freezing-sensitive varieties, respectively [14]. In addition, a previous study found that the semilethal temperature (LT50 values) of “Longyou7” and “Tianyou4” are −5.71 °C and −3.23 °C, respectively [15], which also indicated that freezing tolerance of “Longyou7” was higher than that of “Tianyou4”. The cell membrane integrity, accumulation of osmolytes and activity of antioxidant enzymes are positively correlated with the freezing tolerance of plants [16,17]. EL is a reliable indicator of membrane damage resulting from various stresses [18]. Soluble sugar and proline can maintain the cellular metabolism balance, intracellular homeostasis and stability of cell membranes to improve plant stress tolerance [19,20,21]. In addition, proline over-accumulation can reduce free radical levels [21], and SOD can scavenge excess reactive oxygen species (ROS) against oxidative damage induced by stress conditions [22]. Therefore, these parameters are important biochemical indexes of stress responses. In this study, EL was lower in the roots of “Longyou7” than in “Tianyou4” (Figure 1), indicating that the roots of “Longyou7” experienced less freezing damage than those of “Tianyou4”. The roots of winter turnip rape are important for its survival in winter, and the higher SOD activity, soluble sugar and proline contents might be a reason for the freezing tolerance of “Longyou7”. 

Freezing-induced protein expressional change is an adaptive plant response to freezing stress, which can protect plants against cold injury. Cheng (1992) found that the cold-tolerant wheat variety can induce more proteins than the cold-sensitive variety under cold stress [23]. Here, two winter turnip rape varieties with different freezing tolerances were analyzed under cold-stressed and unstressed conditions by the iTRAQ technique, and we found that there were more up-accumulated proteins in “Longyou7” than in “Tianyou4” (Figure 3A), indicating that these freezing-induced proteins in the roots of “Longyou 7” might be responsible for the improved survival rate under freezing stress. The DAPs induced by freezing stress help to illustrate the freezing tolerance of “Longyou7”. A total of 174 DAPs between two freezing-stressed varieties were identified. A further KEGG analysis revealed that DAPs were involved in important metabolic pathways, which might also be related to the freezing tolerance of “Longyou7”.

### 3.1. Increased Abundance of Ribosome-Related Proteins under Freezing Stress 

Ribosome is composed of small and large subunits, which are the critical sites for protein synthesis. These subunits contain different RNA species and multiple structurally distinct proteins [24]. In this study, a large number of DAPs was involved in Ribosome, including 40S and 60S ribosomal proteins (Figure 6), which play a role in protein synthesis. It is noteworthy that all of these proteins were more abundant in the freezing-stressed “Longyou7” than in the freezing-stressed “Tianyou4” (Table S5, Supplementary Materials). In “Longyou7”, the proteins involved in protein synthesis showed a high accumulation under freezing, which is consistent with the fact that there were more up-accumulated proteins in “Longyou7” than in “Tianyou4” under freezing stress, reflecting that the active protein synthesis of roots is very important for the freezing tolerance of winter turnip rape. In addition, Liu (2017) found that the ribosomal protein was up-accumulated under cold stress, which contributed to the *Flammulina velutipes* mycelia resistance to cold stress [9]. 

### 3.2. Increased Abundance of Proteins Involved in the Biosynthesis of Secondary Metabolites 

The chemical interaction between the plant and the environment is mainly mediated by the biosynthesis of secondary metabolites [25]. Phenylpropanoid is the precursor of various phenolic compounds, with many functions in plants, and is involved in the stress response of plant cells. Lignin is a complex phenolic polymer that increases the strength, rigidity, and hydrophobicity of the plant’s secondary cell wall [26]. Under abiotic stresses, plants can increase the lignin production in specific organs [27,28]. GELP is an important subfamily of lipolytic enzymes involved in secondary metabolite synthesis, defense responses and phenylpropanoid metabolism [29,30]. Peroxidase (POX) plays an essential role in the synthesis of lignin [31]. In this study, up-accumulated peroxidase C3, peroxidase A2 and GELP may contribute to the synthesis of lignin in the roots of freezing-stressed “Longyou7” (Figure 7, Table 1). 

Walls are thin 1 (WAT1) is an integral membrane protein involved in the formation of the secondary cell wall [32]. The mutation of WAT1 in *Arabidopsis* caused a drastic reduction in fiber content and the thickness of the secondary cell wall [33]. Cell wall thickness and lignification could enhance cold tolerance in plants [34]. Compared with the cold-stressed “Tianyou4”, WAT1 was up-accumulated in the cold-stressed “Longyou7” roots (Table 1), indicating that an increased cell wall thickness and lignification could aid the freezing tolerance of “Longyou7”. Previously, we found that the target genes of the cold-stress-responsive miRNAs in “Longyou7”, including *TCP4*, *MYB104* and *ATHB15*, were involved in regulating the cell wall thickness [13]. These genes are essential WAT1-co-regulated genes for either vascular patterning or secondary wall deposition [32].

### 3.3. Decreased Abundance of Energy and Carbohydrate Metabolism-Related Proteins under Freezing Stress

The proteins involved in energy and carbohydrate metabolism are indispensable. Here, DAPs participated in energy and carbohydrate metabolism, including pentose phosphate pathway (PPP), oxidative phosphorylation (OXPHOS), fructose and mannose metabolism, which were down-accumulated in the freezing-stressed “Longyou7”, unlike in “Tianyou4”. Fructose-bisphosphate aldolase (FBPA) is an important enzyme involved in the glycolytic/gluconeogenic pathway and PPP [35]. Down-accumulated FBPA is helpful for the chilling tolerance of plants [36]. Phosphoglucomutase cytoplasmic (cPGM) is involved in sucrose metabolism [37]. The decreased cPGM activity results in increased soluble sugar and glucose contents and improves the cold tolerance of plants [38,39]. Fructokinase (FRK) is important for the phosphorylation of fructose in plants, which can influence the glycolytic pathway and regulate the mutual transformation of soluble sugar and starch [40]. The reduction of FRK1 activity can inhibit the conversion of sucrose to starch [41]. Sucrose is a kind of osmotic substance that can improve the water retention ability of cells under cold stress [42]. In this study, FBPA, cPGM and FRK1 were down-accumulated in “Longyou7” (Figure 8, Table 1), implying that sucrose accumulation plays an important role in the freezing tolerance of “Longyou7”. 

NADH dehydrogenase (ubiquinone) iron–sulfur protein and NADH dehydrogenase (ubiquinone) alpha subcomplex subunit are components of the mitochondrial respiratory chain (electron transport chain) [43]. Thus, down-accumulated NADH dehydrogenase (ubiquinone) iron–sulfur protein and NADH dehydrogenase (ubiquinone) alpha subcomplex subunit can result in a decrease of the respiratory chain activity and sugar consumption in the roots of “Longyou7”. Sugar accumulation is important for the cold tolerance of plants [44,45]. Additionally, V-type proton ATPase subunit proteins (V-ATPase) are essential components of the vacuolar proton pump, which is responsible for the acidification of intracellular compartments and the generation of an electrochemical gradient by hydrolyzing ATP [46]. In *Arabidopsis thalian*a, the V-ATPase protein abundance was significantly increased during CA [47], which was down-accumulated in the freezing-stressed “Longyou7” roots (Figure 8). This difference might result from freezing stress after the induction of CA. However, further analysis should be conducted on the role of V-ATPase in response to freezing stress in the future.

### 3.4. Decreased Abundance of Alpha-linolenic Acid Metabolism-Related Proteins under Cold Stress 

It has been reported that cold-tolerant plants have higher unsaturated membrane lipids [48]. Alpha-linolenic acid, as a structural component of storage and membrane lipids, is a polyunsaturated fatty acid that plays an important role in plant metabolism. Moreover, it is a precursor of the signaling molecule jasmonic acid (JA), which is involved in plant development and stress response [49,50]. Kuiper (1970) found that cold-tolerant alfalfa has a higher unsaturated fatty acid content, especially linolenic acid, than the cold-sensitive variety [51]. Allene oxide cyclase (AOC), glyoxysomal fatty acid beta-oxidation multifunctional protein (MFP) and 12-oxophytodienoate reductase (OPR) are key enzymes of α-linolenic acid metabolism, which can also regulate the synthesis of JA [52]. In this study, these enzymes were down-accumulated under cold stress (Table 1), indicating that the synthesis of JA was inhibited, and a higher linolenic acid content was accumulated in the roots of the freezing-stressed “Longyou7”, compared with that in “Tianyou4”. 

### 3.5. Decreased Abundance of Ascorbate and Aldarate Metabolism-Related Proteins under Freezing Stress 

Ascorbate and aldarate metabolism is an antioxidant defense-related pathway, and monodehydroascorbate reductase (MDAR) participates in the ascorbate-glutathione cycle, a major antioxidant system that protects plants against damage by ROS accumulation [53,54]. Unlike glutathione reductase (GR) and ascorbate peroxidase (APX), little is known about MDAR and its specific isoforms, present in different cell compartments. It has been reported that MDAR has different responses to cold stress. For example, MDAR enzyme activity was increased in cold-stressed rice [55] and scots pine (*Pinus sylvestris*) [56], but the MDAR activity was not affected in cold-stressed *Arabidopsis* [57]. Additionally, MDAR has been reported with functions in other physiological processes related to oxidative stress [58]. In this study, MDAR was down-accumulated in cold-stressed “Lonygou7”, compared with that in “Tianyou4” (Table 1), and further studies are needed to identify the role of MDAR in response to freezing stress in *B. rapa.*

### 3.6. Carbon Fixation-Related Proteins in Freezing-Stressed Winter Rapes

Carbon fixation is the process whereby CO_2_ is incorporated into organic compounds by the Calvin cycle in chloroplasts. Plant roots cannot photosynthesize and fix CO_2_ with a lack of chloroplasts. However, we identified that the ribulose bisphosphate carboxylase large chain (RuBisCO) was down-accumulated in freezing-stressed “Longyou7” (Table 1), which is related to the carbon fixation in photosynthesis [59]. RuBisCO was also found in the seeds of *B. napus* and participated in a new metabolic pathway to increase the efficiency of carbon use during oil biosynthesis [60]. Therefore, the function of RuBisCO in freezing-tolerant “Longyou 7” roots may be considered a novel project for future research.

Overall, we identified and compared freezing-responsive DAPs in the roots of two winter turnip rape varieties using iTRAQ analysis. These data could lay a basis for the identification of DAPs related to freezing tolerance. However, whether these DAPs play key roles in the freezing tolerance of “Longyou 7” is in need of validation. Gene knock-out is the most straightforward and effective method to reveal the molecular function of genes encoding the abovementioned DAPs. Therefore, DAPs, such as up-accumulated peroxidase C3, 60S ribosomal protein L13a-4-like, and down-accumulated FBPA, V-ATPase and RuBisCO under the cold stress of *B. rapa*, will be taken as candidates for future analysis and the molecular dissection of freezing tolerance in winter turnip rape.

## 4. Materials and Methods 

### 4.1. Plant Materials and Freezing Stress Treatment 

“Longyou7” (7R) is a freezing-tolerant winter turnip rape (*B. rapa subsp. rapa*) variety, with a survival rate of more than 90% overwinter at −32 °C, and “Tianyou4” (4R) is a freezing-sensitive winter turnip rape (*B. rapa subsp. rapa*) variety, with a survival rate of 62.4% overwinter at −9 °C [14]. The semilethal temperature (LT50 values) of “Longyou7” and “Tianyou4” are −5.71 °C and −3.23 °C, respectively [15]. The seeds were provided by the College of Agriculture, Gansu Agricultural University (Lanzhou, China). Plants were grown in plastic pots (18 cm in diameter, and 15 cm in depth) filled with a mixture of garden soil and sand (3:1, *w*/*w*) until the six-leaf stage in a greenhouse (20 °C with a 16 h/8 h light/dark cycle), with a photosynthetic active radiation (PAR) of 450 μmol·m^−2^·s^−1^, and then moved into a growth chamber (Safu, Ningbo, China) for pre-CA and freezing treatment. Plants were CA-treated for 48 h at 10 °C and 4 °C separately. After that, plants were freeze treated at −4 °C (the temperature of the mixture of garden soil and sand was −2.4 °C) for 8 h. In the experimental process, the temperature was decreased at a rate of 2 °C·h^−1^. The plants grown at −4 °C were used for freezing treatments (TR), and the untreated plants (20 °C) were used as controls (CK). The lower sections of the root system (Figure S4, Supplementary Materials) of TR and CK were collected, quick-frozen in liquid nitrogen, and stored at −80 °C for protein and RNA extraction. The CK and TR of “Tianyou4” were named 4RCK and 4RTR, respectively. The CK and TR of “Longyou7” were named 7RCK and 7RTR, respectively. 

### 4.2. Analysis of Physiological and Biochemical Parameters

The fresh roots of TR and CK were collected for the determination of biochemical parameters. EL was analyzed according to Huang et al. [61], with slight modifications One gram of the lower section (Figure S4, Supplementary Materials) of the root system of each sample was cut into small fragments (about 2 mm in diameter), washed three times with deionized water, and then placed in 20 mL of deionized water. After 2 h under a shaking condition at room temperature, the initial conductivity (EL1) was determined by a DDS-307 conductivity meter (Leici, Shanghai, China). Then, the root tissue was placed in a boiling water bath for 15 min, and the final conductivity was rerecorded (EL2), after cooling the solution at room temperature. There were 3 replicates per treatment. The relative EL was calculated according to the following formula: 

Relative EL = EL1/EL2 × 100%.

Soluble sugar and proline content was determined following Dubois et al. [62] and Deng et al. [63], respectively. SOD activity was measured by the nitroblue tetrazolium method [64]. These parameters were analyzed by a U-3900H ultraviolet-visible spectrophotometer (Hitachi Limited, Tokyo, Japan), and each treatment was repeated three times.

### 4.3. Protein Extraction and iTRAQ Labeling

iTRAQ analysis was carried out in Beijing Genomics Institute (BGI, Shenzhen, China). The total proteins of the roots from each sample were extracted, according to a previous report [65]. The root tissue from every 10 plants was pooled as one biological replicate, and two biological replicates were included for each sample. The protein concentration was determined by the Bradford assay [66], and protein quality was measured with sodium dodecyl sulfate-polyacrylamide gel electrophoresis (SDS-PAGE). One hundred microgram protein of each sample was digested with 5 μg Trypsin Gold (Promega, Madison, WI, USA) at 37 °C for 16 h and labeled using iTRAQ 8-plex kits (Applied Biosystems, Foster City, CA, USA), following the manufacturer’s instructions. iTRAQ reagents 113 and 114 were used to label the peptides from 4RCK replicates, iTRAQ reagents 116 and 117 were used to label the peptides from 4RTR replicates, iTRAQ reagents 116 and 117 were used to label the peptides from 7RCK replicates, and 119 and 121 were used to label the peptides from 7RTR replicates. The labeled peptide mixtures were pooled and vacuum-dried.

### 4.4. Separation of Peptides and LC-MS/MS Analysis

The peptide mixtures were fractionated on an LC-20AB high-pressure liquid chromatography analyzer (HPLC; Shimadzu, Kyoto, Japan) [65]. The eluted peptides were pooled into 20 fractions, desalted and vacuum-dried. The peptides of each fraction were reconstituted in a solvent consisting of 5% acetonitrile and 0.1% formic acid and centrifuged to remove insoluble impurities at 20,000× *g* for 10 min. The final peptide concentration of each fraction was 0.5 μg/μL. Five microliters of peptides were separated by a 2 cm C18 trap column (inner diameter: 200 μm) in an LC-20AD Nano-HPLC system (Shimadzu, Kyoto, Japan) and then eluted onto a 10 cm analytical C18 column (inner diameter: 75 μm), packed in-house. A Triple TOF 5600 System (AB SCIEX, Concord, ON, Canada) was used to analyze the liquid chromatograph mass spectrometer (LC-MS/MS) of the fractionated samples and acquired data by an electrospray voltage of 2.5 KV [66] at BGI (Shenzhen, China). Information-dependent data acquisition was carried out according to a previous report [67].

### 4.5. iTRAQ Protein Identification and Quantification

A Mascot 2.3.02 search engine (Matrix Science, London, UK) was used for iTRAQ protein identification and quantification. The *B. rapa* protein database 2.0 [68], containing 45,270 sequences, was used as a reference. To reduce the probability of false peptide identification, only peptides found to have a probability of more than 95% by a Mascot probability analysis were counted as identified, and each confident protein identification included at least one unique peptide. DAPs were determined by a permutation test analysis using Bonferroni multiple testing correction. Proteins with a >1.5- or <0.67-fold change and a *p*-value ≤ 0.05 in the two samples were considered as significant DAPs. Protein ratios were quantified by weighing and normalizing them using the median ratio in Mascot. The mass spectrometry proteomics data are available via proteome Xchange, with the identifier, PXD008194. 

The functional category and metabolic pathway of DAPs were analyzed with GO (http://geneontology.org/, Access date: 16th December, 2018) and KEGG (http://www.genome.jp/kegg/, Access date: 16th December, 2018) databases. If the *p*-value was ≤0.05, the GO term or KEEG pathway were regarded as a significant enrichment of differential proteins.

### 4.6. RNA Extraction and qPCR Analysis of Gene Expression 

The total RNA of roots from two varieties under −4 °C (TR) and 20 °C (CK) were isolated using TRNzol Universal Reagent (Tiangen, China), in accordance with the manufacturer’s instructions. First-strand cDNA was synthesized with a SuperScript^®^III RT Reagent Kit (Invitrogen, China, Beijing). qRT-PCR was carried out on a 7900HT Fast Real-Time PCR System (Applied Biosystems, USA), with SYBR qPCR Mix (Invitrogen, California, US). The PCR conditions were followed, as previously reported [13]. The primer sequences for the genes encoding DAPs are listed in Table S6 (Supplementary Materials), and the *β-actin* of *B. rapa* was used as the internal standard. There were three biological replicates, and three technical replicates were performed for each gene. The relative gene expression was calculated using the 2^−ΔΔCt^ method [69].

### 4.7. Data Treatment and Statistical Analysis

For the data of the physiological parameters and qPCR analysis, the mean and SD were calculated from three repeats of each treatment, and the differences were analyzed by Duncan’s multiple range test (*p* < 0.05) and an independent-samples *t*-test (*p* < 0.05). 

## 5. Conclusions

In this study, iTRAQ-based proteomic technology was used to investigate the differential proteomics of winter turnip rape roots under cold stress, and 139 and 96 DAPs were identified in “Longyou7” and “Tianyou4”, respectively. In addition, 174 DAPs were identified between two freezing-stressed varieties. Based on the functional analysis, we concluded that the increased ribosome and biosynthesis of secondary metabolites, decreased energy and carbohydrate metabolism via PPP, OXPHOS, fructose and mannose metabolism could improve protein synthesis, the cell wall thickness of roots and the accumulation of sugar, which are conducive to the survival of winter turnip rape under freezing stress. Moreover, the accumulation of linolenic acid caused by a decrease in alpha-linolenic acid metabolism can be regared as a reason for the freezing tolerance of winter turnip rape. In summary, these findings increased our understanding of the molecular mechanisms involved in freezing tolerance in winter turnip rape.

## Figures and Tables

**Table 1:** Representation of DAPs in two varieties of winter turnip rape under freezing stress.

Uniprot ID	Protein	Fold Changes ^a^		
		7RTR/7RCK Fold Change	4RTR/4RCK Fold Change	7RTR/4RTR Fold Change
PSB2A_ARATH	Proteasome subunit beta type-2-A	2.028884928	-	1.716545467
ECH2_ARATH	Enoyl-CoA hydratase 2, peroxisomal	0.636897776	-	-
SPD1_ARATH	Spermidine synthase 1	1.736926897	-	-
NOP56_SCHPO	Nucleolar protein 56	1.801418945	-	-
LOX2_ARATH	Lipoxygenase 2, chloroplastic	2.921783613	-	-
P5CS2_ARATH	Delta-1-pyrroline-5-carboxylate synthase B	1.957524834	-	-
CBF5_ARATH	H/ACA ribonucleoprotein complex subunit 4	1.995205069	-	-
SAHH1_ARATH	Adenosylhomocysteinase 1	-	1.75364388	-
BAM5_ARATH	Beta-amylase 5	-	0.52337023	2.646139818
PGIP1_ARATH	Polygalacturonase inhibitor 1	-	0.61557221	-
PER17_ARATH	Peroxidase 17	-	0.61557221	-
UGDH3_ARATH	UDP-glucose 6-dehydrogenase 3	-	1.54961483	-
GDL20_ARATH	GDSL esterase/lipase	-	0.46826289	1.695003118
VATB3_ARATH	V-type proton ATPase subunit B3	-	-	0.553787078
ALF_CICAR	Fructose-bisphosphate aldolase	-	-	0.502403227
MDAR3_ARATH	Probable monodehydroascorbate reductase	-	-	0.478651486
NDUA9_ARATH	Nicotinamide adenine dinucleotide (NADH) dehydrogenase [ubiquinone] 1 alpha subcomplex	-	-	0.608297667
NDUS1_ARATH	NADH dehydrogenase [ubiquinone] iron-sulfur protein 1	-	-	0.551175594
SCRK1_ARATH	Probable fructokinase-1	-	1.55896	0.629339177
RS3A2_ARATH	40S ribosomal protein S3a-2	-	-	1.624504793
PERA2_ARMRU	Peroxidase A2	-	-	1.990433075
AOC4_ARATH	Allene oxide cyclase 4	-	-	0.566248831
RBL_BRAOL	Ribulose bisphosphate carboxylase large chain	-	0.55295901	0.510849202
PGMC1_ARATH	Probable phosphoglucomutase, cytoplasmic 1	0.662925032	-	0.545940075
PER3_ARMRU	Peroxidase C3	1.622786225	-	2.427233592
R13A4_ARATH	60S ribosomal protein L13a-4	-	-	2.828427125
RS3A_BRACM	40S ribosomal protein S3a	-	-	1.686837421
RS262_ARATH	40S ribosomal protein S26-2	-	-	1.688842594
WAT1_ARATH	Protein WALLS ARE THIN 1	-	-	1.684822612
MFPA_BRANA	Glyoxysomal fatty acid beta-oxidation multifunctional protein MFP-a	-	-	0.543328591
MLP34_ARATH	MLP-like protein 34	-	-	1.512082689
MDAR3_ARATH	Monodehydroascorbate reductase	-	-	0.478651486
OPR1_ARATH	12-oxophytodienoate reductase 1	-	-	0.509956713
ALF_CICAR	Fructose-bisphosphate aldolase	-	-	0.451437041

^a^ Fold changes; “-” means no differential accumulation detected.

## Supplementary Materials

Supplementary materials can be found at http://www.mdpi.com/1422-0067/19/12/4077/s1.

## Abbreviations

iTRAQ	isobaric tags for relative and absolute quantification
2DE	two-dimensional electrophoresis
EL	electrolyte leakage
SOD	superoxide dismutase
APX	ascorbateperoxidase
TEAB	triethylamonium bicarbonate
CK	control
TR	treatment
DAPs	differently accumulated proteins
GO	gene ontology
KEGG	Kyoto Encyclopedia of Genes and Genomes
qRT-PCR	quantitative real-time polymerase chain reaction
ROS	reactive oxygen species
FBPA	fructose-bisphosphate aldolase
cPGM	probable phosphoglucomutase cytoplasmic
FRK	fructokinase
POX	peroxidase
GELP	GDSL esterase/lipase-like protein
V-ATPase	V-type proton ATPase subunit proteins
P5CS	delta-1-pyrroline-5-carboxylate synthase
JA	jasmonic acid
AOC	allene oxide cyclase
MFP	glyoxysomal fatty acid beta-oxidation multifunctional protein
WAT1	protein walls are thin 1
OPR	12-oxophytodienoate reductase
MDAR	monodehydroascorbate reductase
MLP	major latex-like protein
GR	glutathione reductase
RuBisCO	ribulose bisphosphate carboxylase large chain
