# Supplementary figures and images for: iTRAQ-Based Comparative Proteomic Analysis of the Roots of TWO Winter Turnip Rapes (Brassica rapa L.) with Different Freezing-Tolerance
Source: Int J Mol Sci. 2018 Dec 17;19(12):4077. doi: 10.3390/ijms19124077 (PMC6321220; doi:10.3390/ijms19124077)

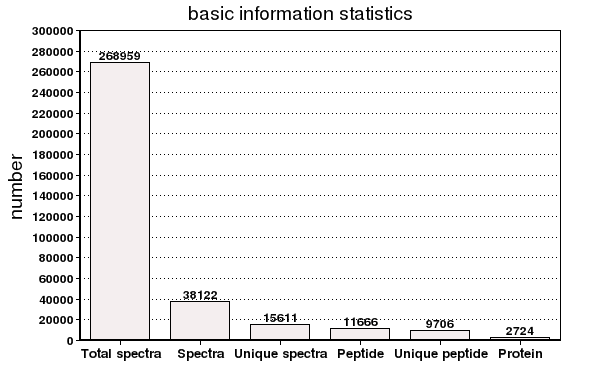

Supplement: Supplementary file 1 [file ijms-19-04077-s001.zip › Supplementary files- ijms-387367/Fig S1.png]

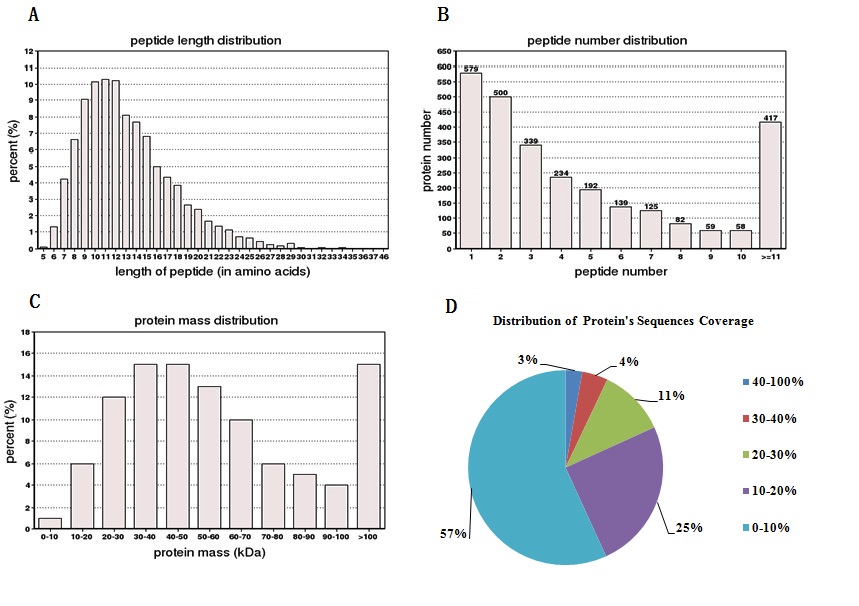

Supplement: Supplementary file 1 [file ijms-19-04077-s001.zip › Supplementary files- ijms-387367/Fig S2.jpg]

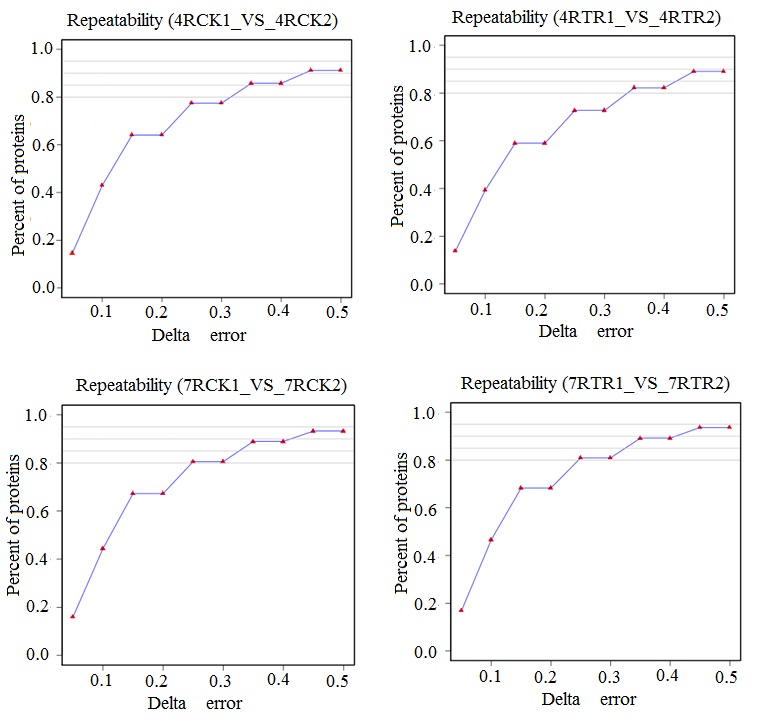

Supplement: Supplementary file 1 [file ijms-19-04077-s001.zip › Supplementary files- ijms-387367/Fig S3.jpg]

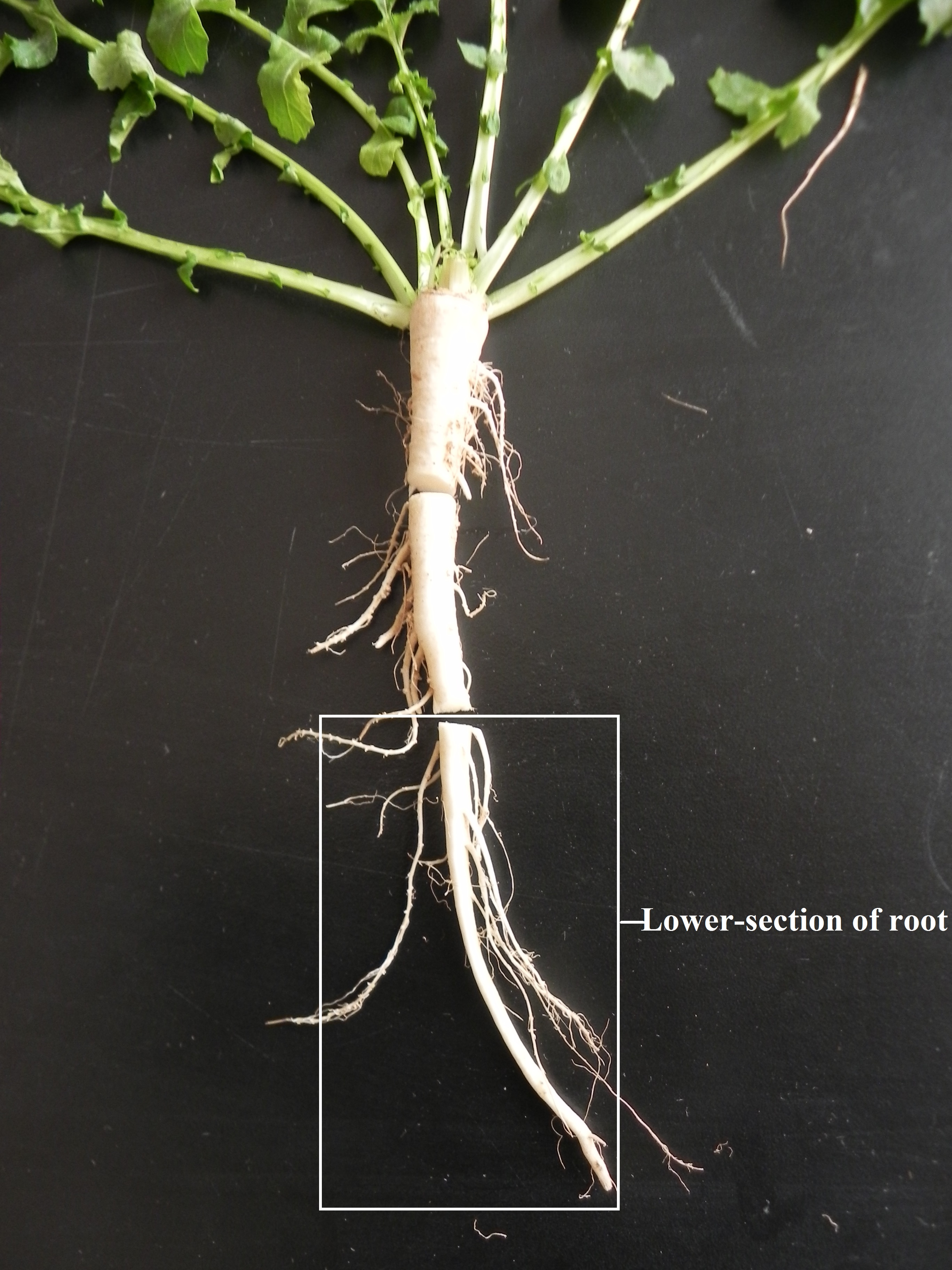

Supplement: Supplementary file 1 [file ijms-19-04077-s001.zip › Supplementary files- ijms-387367/Fig S4.jpg]
